# The Adaptive Remodeling of Endothelial Glycocalyx in Response to Fluid Shear Stress

**DOI:** 10.1371/journal.pone.0086249

**Published:** 2014-01-20

**Authors:** Ye Zeng, John M. Tarbell

**Affiliations:** 1 Department of Biomedical Engineering, The City College of New York, New York, New York, United States of America; 2 Institute of Biomedical Engineering, School of Preclinical and Forensic Medicine, Sichuan University, Chengdu, China; Cardiological Center Monzino, Italy

## Abstract

The endothelial glycocalyx is vital for mechanotransduction and endothelial barrier integrity. We previously demonstrated the early changes in glycocalyx organization during the initial 30 min of shear exposure. In the present study, we tested the hypothesis that long-term shear stress induces further remodeling of the glycocalyx resulting in a robust layer, and explored the responses of membrane rafts and the actin cytoskeleton. After exposure to shear stress for 24 h, the glycocalyx components heparan sulfate, chondroitin sulfate, glypican-1 and syndecan-1, were enhanced on the apical surface, with nearly uniform spatial distributions close to baseline levels that differed greatly from the 30 min distributions. Heparan sulfate and glypican-1 still clustered near the cell boundaries after 24 h of shear, but caveolin-1/caveolae and actin were enhanced and concentrated across the apical aspects of the cell. Our findings also suggest the GM_1_-labelled membrane rafts were associated with caveolae and glypican-1/heparan sulfate and varied in concert with these components. We conclude that remodeling of the glycocalyx to long-term shear stress is associated with the changes in membrane rafts and the actin cytoskeleton. This study reveals a space- and time- dependent reorganization of the glycocalyx that may underlie alterations in mechanotransduction mechanisms over the time course of shear exposure.

## Introduction

The endothelial glycocalyx, which is localized on the luminal surface of the endothelium, interacts directly with blood flow, and plays important roles in endothelial mechanotransduction [1]–[3], modulation of vascular permeability [4], [5], and mediation of leukocyte adhesion [2], [6]. Our previous study demonstrated that initial exposure of statically cultured endothelial cells (ECs) to a step change in shear stress induced dramatic reorganization of the glycocalyx within 30 min [7]. In the present study, we investigated the adaptive remodeling of endothelial glycocalyx to long-term (24 h) fluid shear stress exposure that should more faithfully represent the condition of fully adapted endothelial cells in vivo.

The endothelial glycocalyx contains various proteoglycans (PGs) with associated glycosaminoglycans (GAGs), such as heparan sulfate (HS) and chondroitin sulfate (CS). Syndecan-1 and glypican-1 are two major heparan sulfate proteoglycans (HSPGs) on apical EC surfaces. Syndecan-1 possesses both HS and CS chains while glypican-1 exclusively carries HS chains [8], [9]. Syndecan-1 is localized on the plasma membrane via a single transmembrane domain [10] that interacts with cytoskeleton. Glypican-1 is anchored to membrane rafts (MRs) by a glycosylphosphatidylinositol (GPI) linkage [11]–[13].

MRs are classified into two types: protein-based membrane domains (i.e., caveolae) and lipid-based domains (i.e., lipid rafts) [7], [14]. The cholera toxin B subunit (CTx-B), which binds specifically to a component of the plasma membrane–glycosphingolipid, ganglioside GM_1_, has been used as a MR marker in many studies [15], [16]. Caveolin-1, which anchors caveolae to the actin cytoskeleton [17], has emerged as a vital plasma membrane mechanosensor [18]. Meanwhile integrity of the actin cytoskeleton is essential for the immobility of caveolae [19]. In contrast, lipid rafts are held together by specific lipid-lipid interactions [20], organized in a liquid-order phase, and characterized by limited acyl-chain order but high translational mobility [14], [21].

Adaptation of the endothelium to fluid shear stress is dominated by transformation in the actin cytoskeleton resulting in rearrangement of filamentous actin (F-actin) into bundles of stress fibers aligned in the direction of flow and into a diffuse network of short microfilaments including lamellipodia and filopodia [22]–[24]. The stress fiber bundles are composed of actin filaments in parallel alignment that function as cellular cytoskeletal-contractile elements [23], [25]. In static conditions (no shear stress), prominent microfilament bundles, the dense peripheral bands, are present at the cell periphery of confluent EC monolayers [25], [26].

Previous work in our laboratory and others demonstrated that HS plays a central role in mediating fluid shear stress-induced cell motility and proliferative response [27], and change of the actin cytoskeleton [28], [29]. Our recent study showed that 15 dyn/cm^2^ of shear stress induced the junctional clustering of HS via mobility of GPI-anchored glypican-1 in lipid rafts during the initial shear exposure up to 30 min. In contrast, the transmembrane protein syndecan-1 with attached HS and CS, seemed to be fixed in position, as did the fraction of glypican-1 with attached HS bound to caveolae [7].

In the present study, we further investigated changes of the glycocalyx PGs with their associated GAGs, MRs (caveolin-1 and GM_1_), and the spatial distribution of the actin cytoskeleton under fluid shear stress for an extended time (24 h). Our results show that new synthesis of glypican-1, syndecan-1 and HS converge to restore the uniform distribution of HS over the cell surface that was present at time 0. Shear-induced increases in caveolin-1/caveolae and actin are predominantly distributed in the apical regions of the cell where a sustained clustering of lipid rafts occurs. We conclude that adaptation of the glycocalyx to long-term shear stress is associated primarily with new synthesis of its components and changes in organization of MRs and the actin cytoskeleton.

## Materials and Methods

### Cell Culture

The rat fat pad endothelial cell (RFPEC), a well-defined cell culture model for studying the effect of shear stress on the glycocalyx [7], [28], was cloned from cells originally isolated from rat epididymal fat pad (a gift from Drs. David C. Spray and Mia M. Thi, Albert Einstein College of Medicine, Bronx, NY) [30] and cultured in the present study as described previously [7], [28], [31].

Bovine aortic endothelial cells (BAEC) were purchased from VEC Technologies and grown in MEM supplemented with 10% FBS, 1% L-glutamine, and 1% penicillin-streptomycin [32]. Cells (passage 6) were plated on to 50 µg fibronectin pre-coated glass slides at a density of 1×10^5^ cells/cm^2^ and cultured for 4–6 days until they attained confluence.

### Shear Application

The shear stress was initiated by a step change from zero to 15 dyn/cm^2^ using a parallel-plate flow chamber as described previously [7], [33], [34], and was applied on RFPEC or BAEC monolayers. Briefly, the shear stress reached its steady state value rapidly after we turned on the pump which maintained the hydraulic pressure in the flow system. The circulating medium for shearing flow was DMEM (for RFPEC) or MEM (for BAECs) with 5% FBS and 0.5% BSA unless indicated otherwise. The flow system was kept at 37°C in a humidified 5%/95% CO_2_/air incubator. The cell morphology was visualized using a Nikon Eclipse TE2000-E inverted microscope (Nikon) with a digital camera (Photometrics cascade 650, Roper Scientific).

### Cytochalasin D Treatments

The role of the actin cytoskeleton in the shear stress-induced adaptive remodeling of the glycocalyx was further investigated using an inhibitor of actin polymerization, cytochalasin D (CD, Sigma). The optimal concentration of CD (40 nM) was determined by evaluating its dose-dependent effects on EC viability, morphology, and actin cytoskeleton. For shear experiments, cells were pre-incubated with 40 nM CD for 1 hour and were subsequently subjected to shear stress in circulating medium with the same concentration of CD (40 nM).

### Immunofluorescence Staining

Immediately after exposure to shear stress, the distributions of glycocalyx components (HS, CS, glypican-1 and syndecan-1), and membrane rafts (caveolin-1 and GM_1_) were detected using immunofluorescence staining methods as described previously [7]. For visualization of the actin cytoskeleton, after shear exposure, cells were fixed in 2% PFA, permeabilized with 0.1% Triton X-100, and stained with Alexa Fluor 488 phallotoxin (1 unit per coverslip; Molecular Probes) for 20 min. Negative controls were carried out by omitting primary antibodies or binding proteins. All images shown in the present paper had the background subtracted unless indicated otherwise.

### Confocal Microscopy and Quantification Analysis

All samples were imaged with a Zeiss LSM 510 laser scanning confocal microscope (Confocal Microscopy Laboratory, The City College of New York) using a Plan-Apochromat 63×/1.4 Oil DIC objective as described previously [7], [31]. The image stacks were analyzed with ImageJ software (version 1.46; NIH), and the mean fluorescence intensity (MFI, mean±SE) and coverage were assessed using the max-intensity Z-projection images as described previously [7], [31].

For distribution analysis, the boundary of each cell was outlined by the ImageJ polygon selection tool. Then, the radial profile plug-in automatically changed the borders to the best-fit circles for subsequent radial distribution analysis [7]. Because the morphology of BAEC was fusiform after 24 h shear stress application, we obtained and normalized the intensities of pixels along lines from the centroid to boundary points, and then developed a new scattering distribution method to examine the distribution of HS on these cells.

A new vertical (Z) spatial distribution analysis method was used for caveolae and actin. In brief, a plane crossing the center of the cell in Z direction was selected as the interface. The images above or below the interface were extracted from the original Z-stack to make two new substacks in the same order, which were defined as the apical stack and basal stack, respectively. Then, the MFIs and coverages of these stacks were assessed using the max-intensity Z-projection images of the substacks.

### Statistical Analysis

Data are presented as mean±SD obtained from at least three independent experiments (n = 3) unless indicated otherwise. The images for coverage calculations were obtained from at least 12 max-intensity Z-projection images. At least 40 cells were chosen for radial distribution analyses, and 10 cells were chosen for scattering distribution analyses. Statistical analysis was performed by one-way analysis of variance (ANOVA) with either the least significant difference (LSD) test or Tamhane’s T^2^ test (depending on Levene’s statistic for homogeneity of variance), and nonparametric tests (Wilcoxon Paired Signed-Rank Test) using the SPSS 20.0 software package. Differences in means were considered significant if *P*<0.05.

## Results and Analysis

### Redistribution of GAG Under Long-term Shear Stress

#### The restoration of HS

In our previous study [7], we showed that HS covered **most** of the apical cell surface under static conditions, and moved toward the cell’s downstream edge during 10 min of 15 dyn/cm^2^ shear stress application, and eventually clustered at the cell boundary after 30 min.

**Figure 1:**
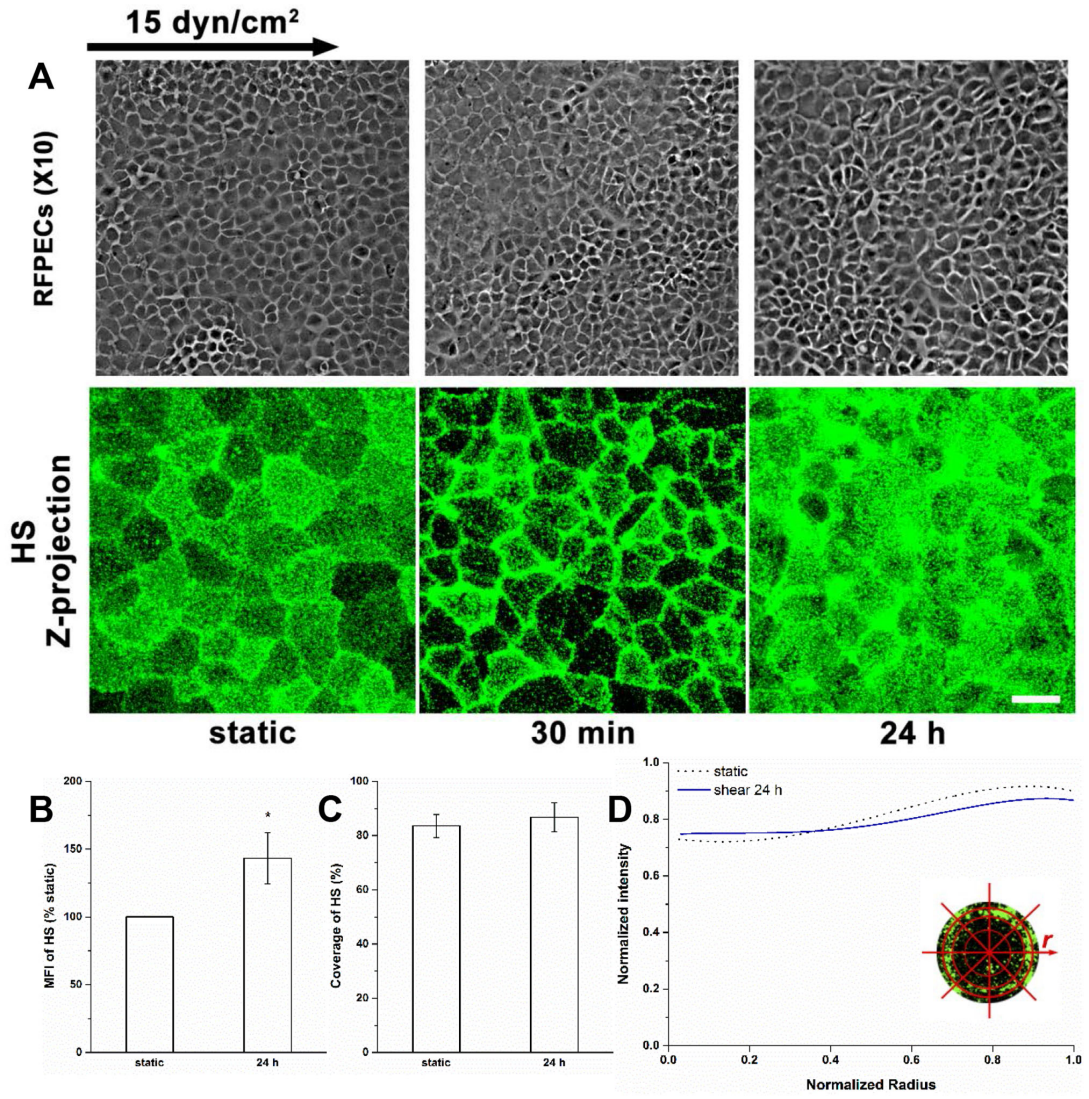
Restoration of HS after 24 h of shear exposure. (A) Top: Phase contrast micrographs of confluent RFPEC monolayers show that they fail to undergo change in cell shape under shear stress. Bottom: Representative immunofluorescent images of HS under static and shear stress conditions showing boundary clustering at 30 min and restoration of coverage at 24 h. (B) MFI (n = 16 images), (C) Coverage, and (D) Radial distribution of HS. The zero-radius represents the center of cell. The boundary of each cell was outlined by ImageJ. Then, the radial profile plug-in automatically changed the borders to the best-fit circles (D inset) for subsequent analyses. Scale bar: 20 µm. *P<0.05; **P<0.01.

The redistribution of HS after long-term shear exposure was further investigated (Fig. 1). After 24 h of shear exposure, HS was reclaimed in the central region of the cell surface, but the clustering HS was still observable at the cell boundary (Fig. 1A and D). The MFI of HS showed a significant increase about 43% above static (*P*<0.05, Fig. 1B). The coverage of HS was restored to 86.8±5.4%, the same level as static condition (Fig. 1C). The distribution of HS was returned to nearly uniform (Fig. 1D). These results suggest that new synthesis of HS contributes to the restoration of HS in the central region of the cell.

**Figure 2:**
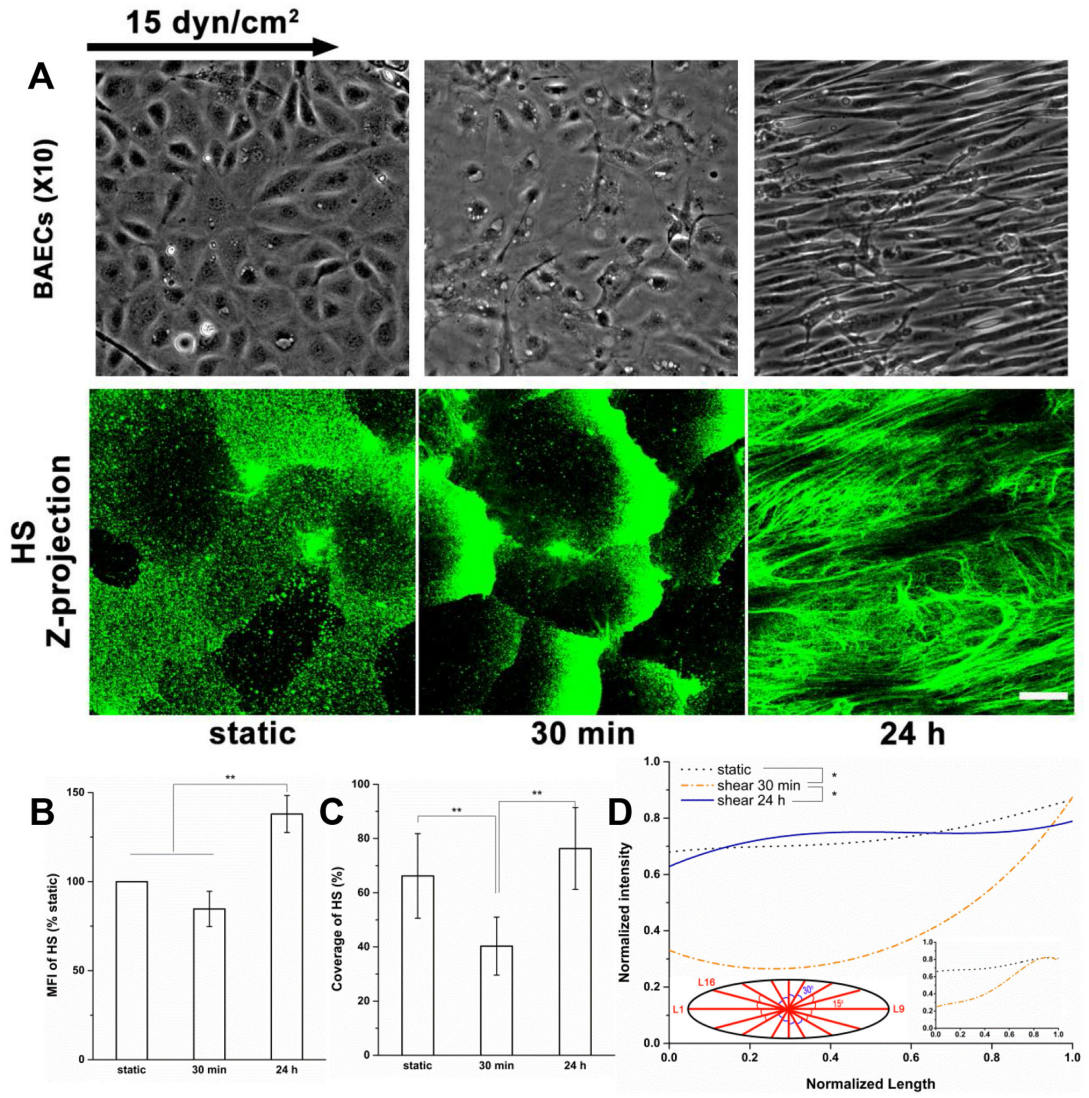
Validation of the clustering and subsequent restoration of HS under shear stress using bovine aortic endothelial cells. (A) Top: Phase contrast micrographs of confluent BAEC monolayer reveal a typical dynamic change in cell morphology from cobblestone (static control) to the elongated (fusiform) and oriented in the direction of flow. Bottom: Representative immunofluorescent images of HS under static and shear stress conditions. (B) MFI, (C) Coverage, and (D) Scattering distribution displays the average intensity along lines from the centroid to the boundary of cells (n = 10 cells). For each cell, 16 lines (D left insert) were selected to analyze the scattering distribution. We also showed the radial distributions (D right insert, plotted by normalized radius on the horizontal axis and normalized intensity on the vertical; n = 50 cells). No significant differences were found between radial distribution and scattering distribution at both static and 30 min (*P*>0.05) indicating the radial distribution is appropriate for cobblestone ECs. The clustering of HS at 30 min and restoring of HS at 24 h on BAECs are similar to the results on RFPECs showed in our previous [7] and the present study, respectively. Scale bar: 20 µm. *P<0.05; **P<0.01.

Similar phenomena were observed on BAECs during shear exposure (Fig. 2). BAECs maintained the cobblestone shape at 30 min, but after 24 h, the BAECs became fusiform and oriented to the direction of flow (Fig. 2A). HS clustered at the cell boundary at 30 min, and was restored in the central region of the cell surface after 24 h (Fig. 2A and D) with a higher MFI of 138% of static (*P*<0.05; Fig. 2B) and an insignificant increase in coverage (*P* = 0.17; Fig. 2C). The scattering distribution (Fig. 2D) showed the average intensity along lines from the centroid to the boundary of cells (Fig. 2D left insert). At 30 min, HS was concentrated at the cell boundary. After 24 h, the distribution of HS returned to the static level. These results further confirmed our observation of the adaptive remodeling of HS on the surface of endothelial cells.

No significant difference was found between the distributions of HS obtained by using the radial distribution (Fig. 2D right insert) and the scattering distribution at both static and 30 min (*P* = 0.23) indicating that both the radial distribution and the scattering distribution are appropriate for cobblestone ECs.

#### The synthesis of CS

**Figure 3:**
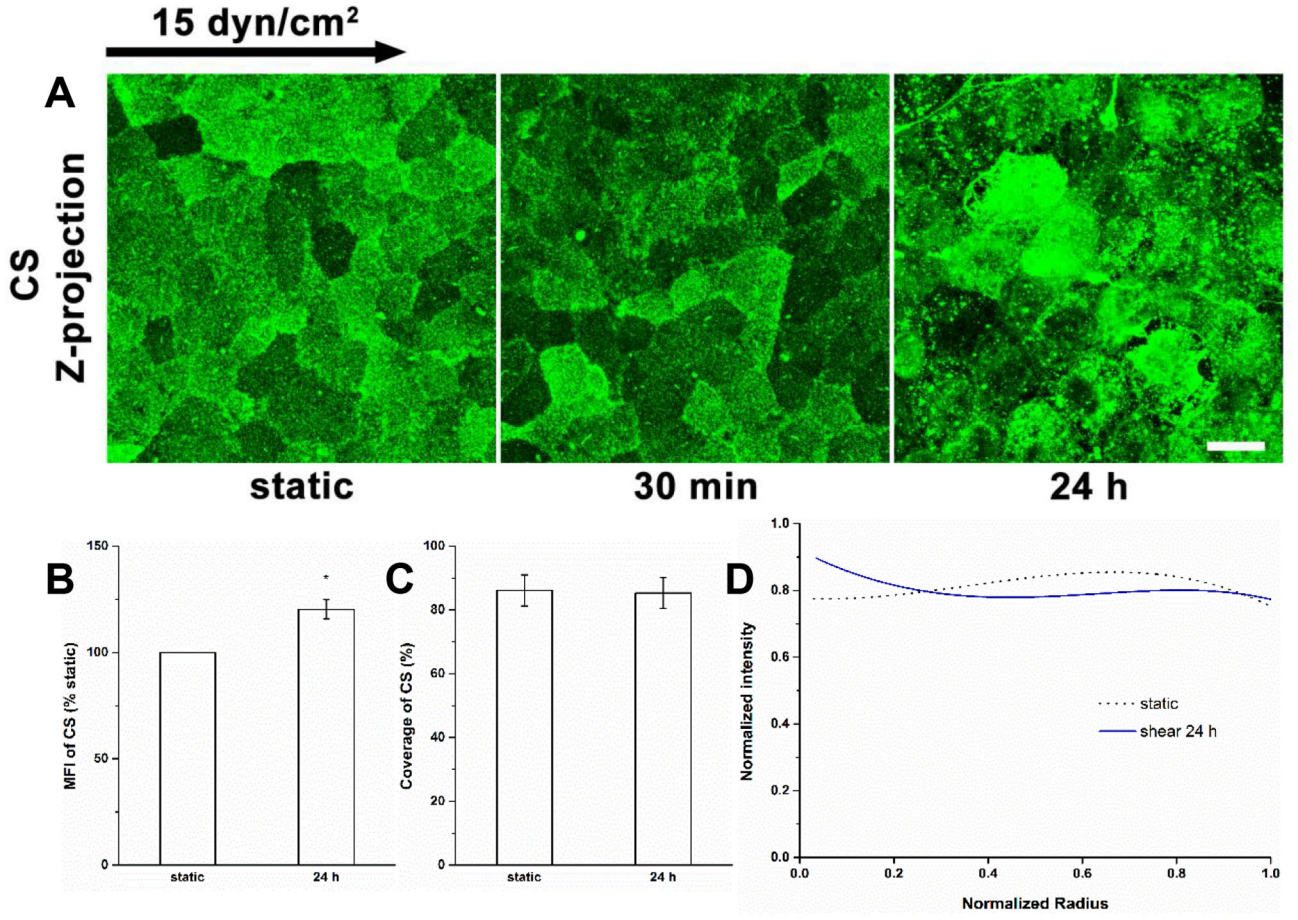
Redistribution of CS after 24 h of shear exposure. (A) Immunofluorescent images of CS under static condition and shear exposure for 30 min and 24 h. (B) MFI, (C) Coverage, and (D) Radial distribution of CS. Scale bar: 20 µm. *P<0.05.

Unlike HS, the MFI, coverage and distribution of CS did not change significantly when cells were exposed to shear stress for 30 min as previously described [7]. After 24 h (Fig. 3), CS at cell-cell appositions was slightly disrupted (Fig. 3A). The MFI of CS increased significantly by nearly 20% of static (Fig. 3B), although the coverage of CS remained at 85.3±4.9%, close to the static level (Fig. 3C), and the distribution did not change significantly (Fig. 3D). Therefore, long-term shear stress induces the synthesis of CS with a nearly uniformly distribution on the apical cell surface.

### Redistribution of Glypican-1

**Figure 4:**
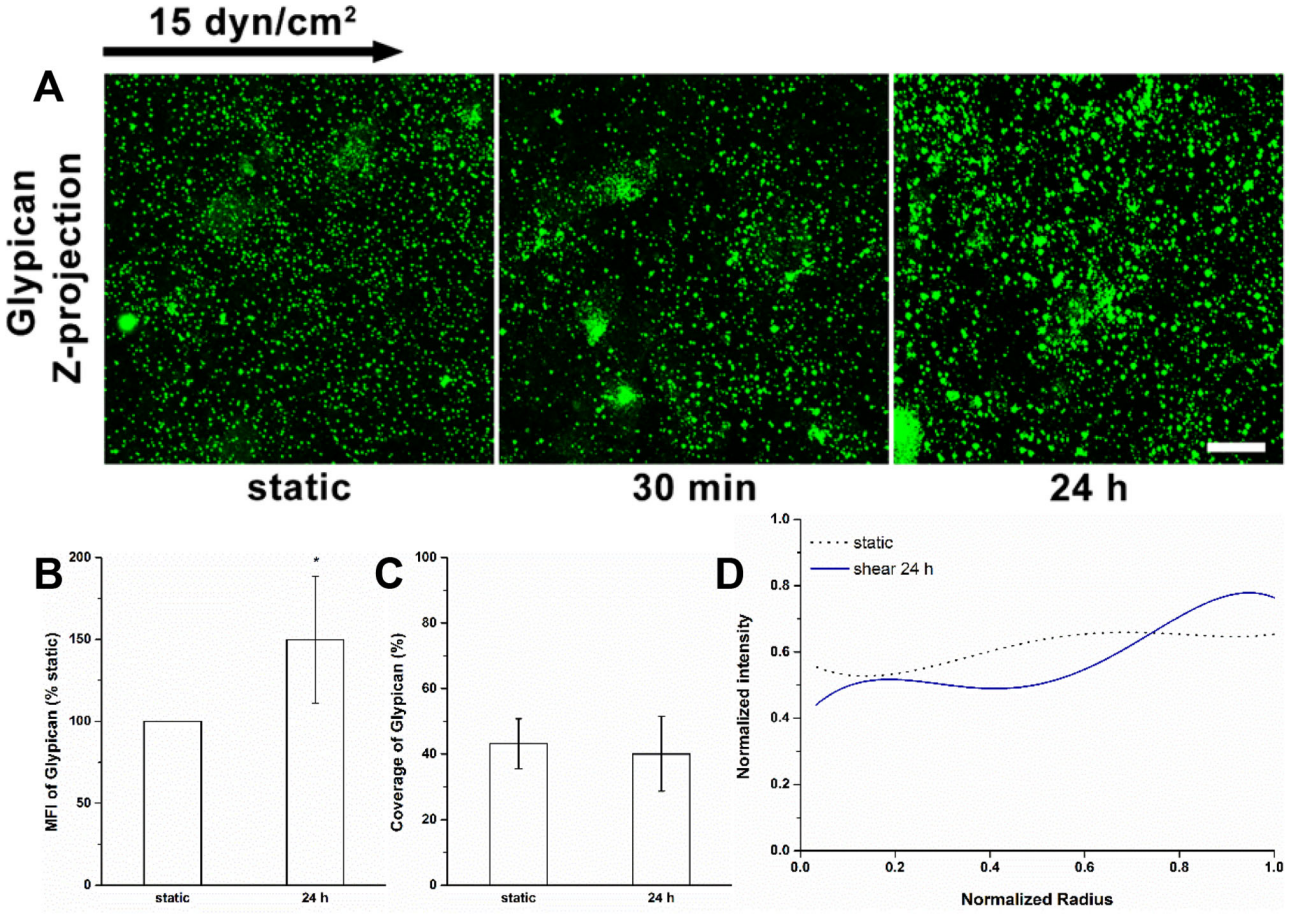
Redistribution of glypican-1 under shear stress. (A) Immunofluorescent images show that shear stress induces clustering of glypican-1 at 30 min, and enhances the glypican-1 intensity at 24 h. (B) MFI, (C) Coverage, and (D) Radial distribution of glypican-1 (n = 80 cells). Scale bar: 20 µm. *P<0.05; **P<0.01.

The coverage of glypican-1 on the apical surface decreased by nearly 49% during 30 min of shear exposure as previously described [7], then increased to the baseline level again after 24 h of exposure (Fig. 4A and C). A 50% increase in the glypican-1 MFI was observed at 24 h (Fig. 4B). There was no difference in the distribution of glypican-1 at 24 h compared to the static condition (Fig. 4D). Although glypican-1 continued to cluster in the cell boundary at 24 h, the distribution over the cell surface was more even than at 30 min (*P*<0.05). These observations support a sustained clustering of glypican-1 and significant synthesis on the cell surface.

### The Synthesis of Syndecan-1

**Figure 5:**
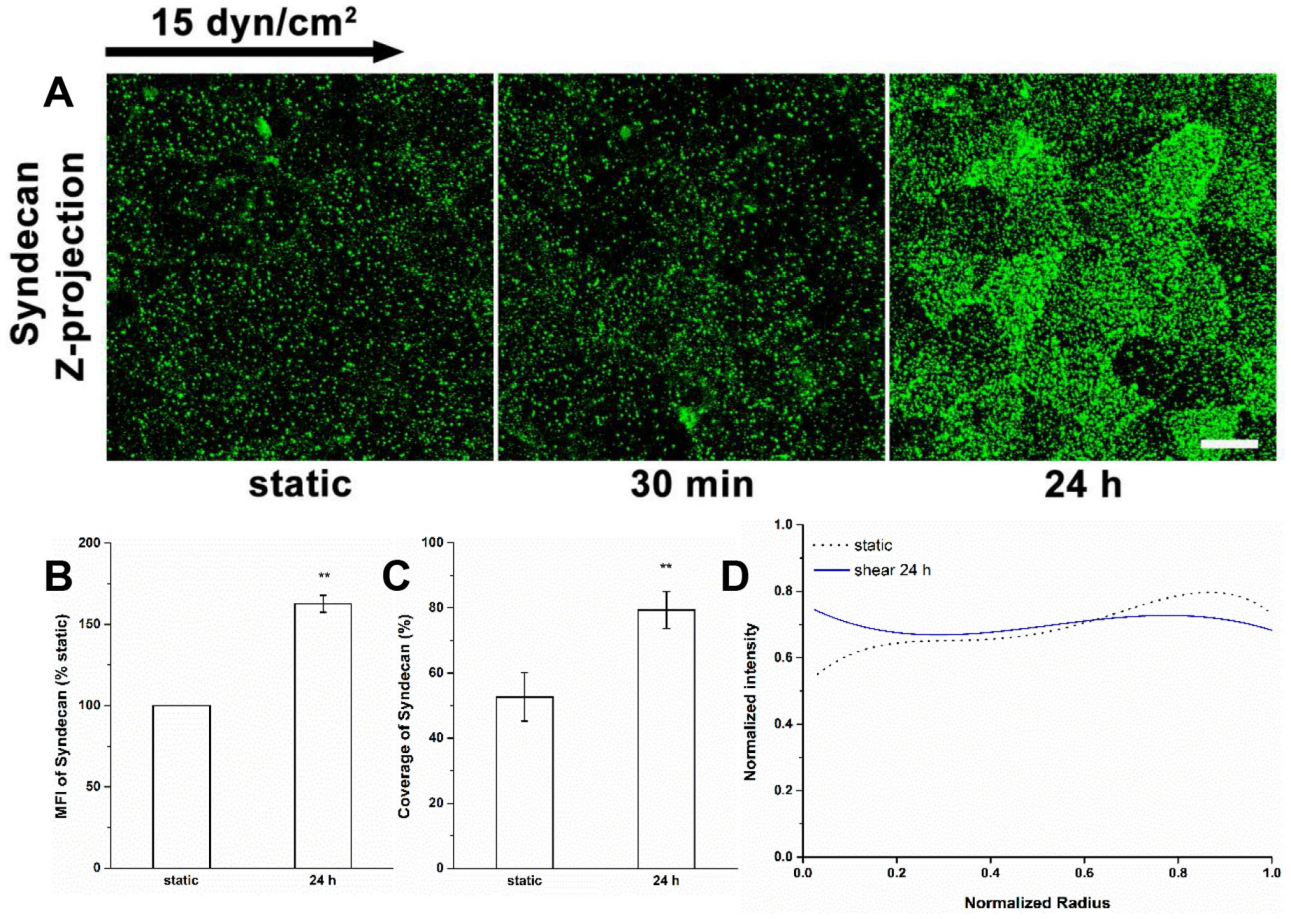
Synthesis and distribution of Syndecan-1 under shear stress. (A) Immunofluorescent images show that shear stress induces the syndecan-1 increase after 24 h of shear exposure. (B) MFI, (C) Coverage, and (D) Radial distribution of syndecan-1. Scale bar: 20 µm. **P<0.01.

During the first 30 min of shear exposure, the MFI, coverage, and distribution of syndecan-1 did not change, as previously described [7] (Fig. 5). After exposure to shear stress for 24 h, the MFI of syndecan-1 increased by nearly 63% (Fig. 5A and B), the coverage was raised to 79.4±5.7% (vs. 52.7±7.4% at static condition, *P*<0.01; Fig. 5C). But the distribution of syndecan-1 under all of these conditions was stable (Fig. 5D). The increases in MFI and coverage of syndecan-1 without alteration of the distribution, suggest that shear stress induced the synthesis of syndecan-1 that was evenly distributed over the cell surface.

### The Spatial Distribution of Caveolae/caveolin under Shear Stress

**Figure 6:**
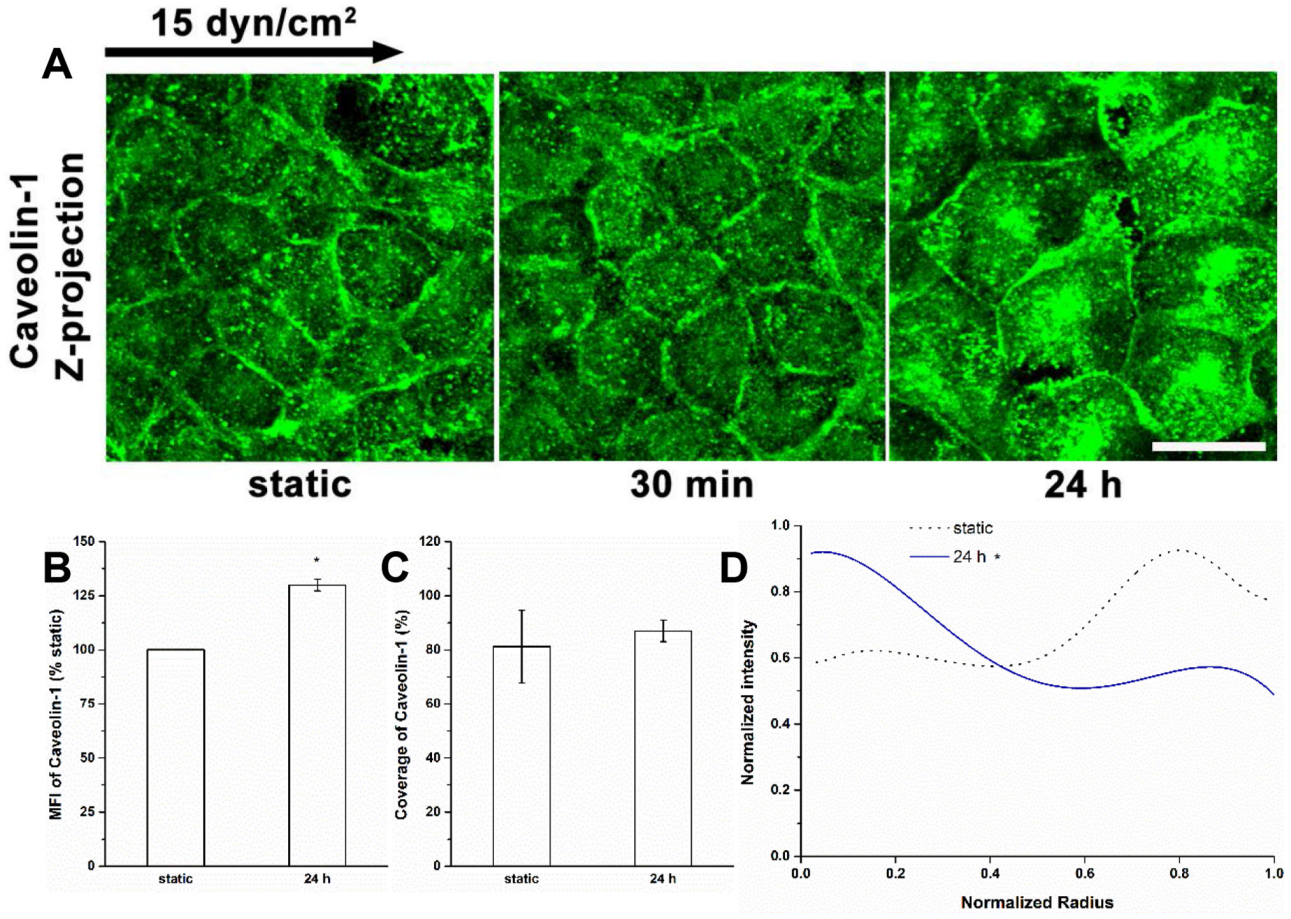
Redistribution of caveolae/caveolin under shear stress. (A) Immunofluorescent images of caveolin-1 at time points. (B) MFI, (C) Coverage, and (D) Radial distribution of caveolin-1. After 24 h exposure to shear stress, the MFI of caveolin-1 was increased, and its localization at the center of cells was enhanced. Scale bar: 20 µm. *P<0.05.

**Figure 7:**
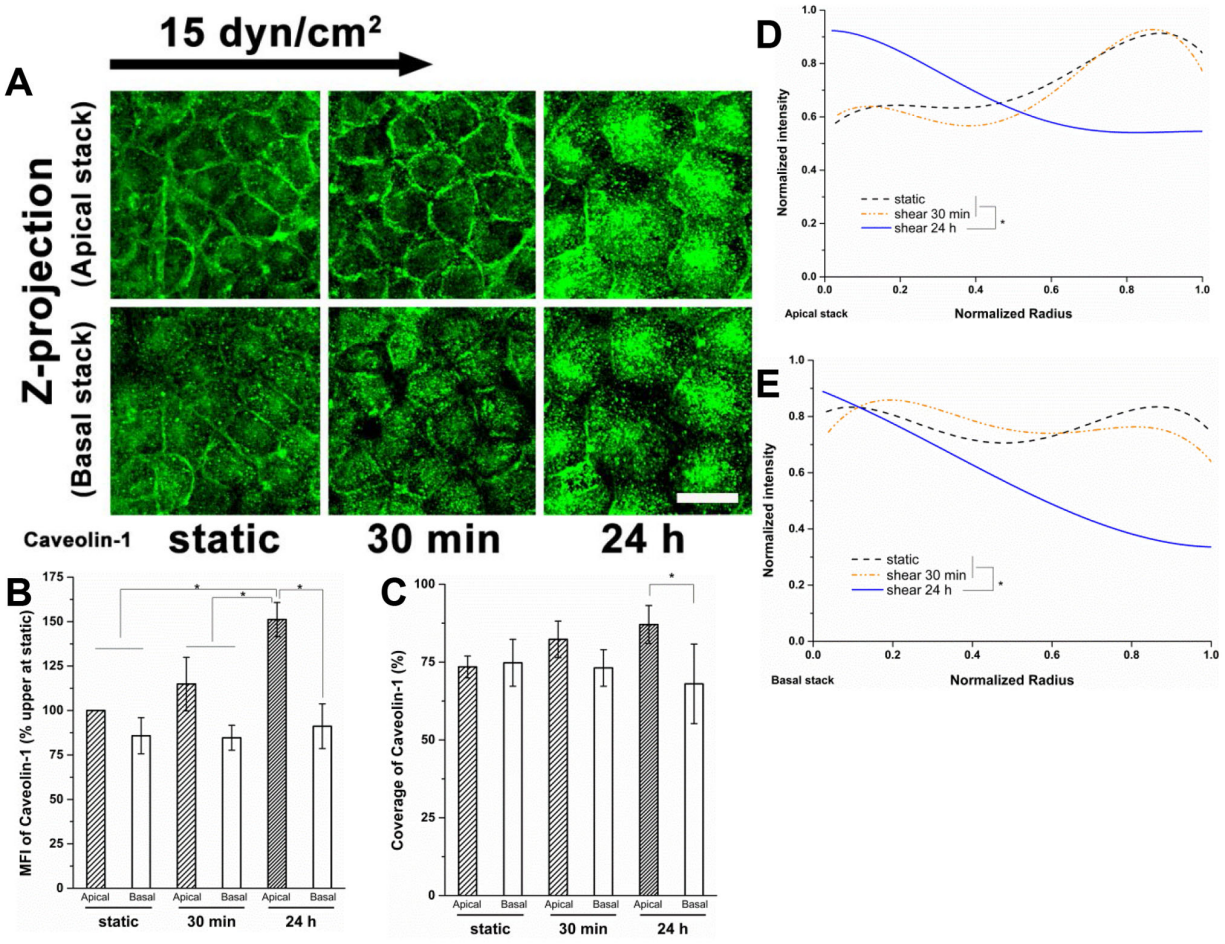
The vertical spatial distribution of caveolae/caveolin under shear stress. (A) Z-projection of the apical and basal stack. The interface between two substacks is the surface (layer) crossing the center of the cell edges. (B) MFI, (C) Coverage, (D) radial distribution in the apical stack and (E) the basal stack. Much more caveolin-1 was distributed in the apical stack than in the basal stack under shear stress, especially for 24 h. In the apical stack, the caveolin-1 concentrated more near the cell boundary under the static condition, and moved to the cell interior after exposure to shear stress for 24 h; in the basal stack, the caveolin-1 was distributed nearly uniformly under static conditions and at 30 min; the caveolin-1 near the cell boundary decreased after 24 h. Scale bar: 20 µm. *P<0.05.

Figures 6 and 7 show a time-dependent spatial re-distribution of caveolin-1 in ECs exposed to fluid shear stress. The caveolin-1 was well expressed at all time points (Fig. 6A). The MFI did not change in cells exposed to the flow for 30 min as previously described [7], but increased significantly by about 30% after 24 h of exposure without coverage change (Fig. 6B and C). During the first 30 min, like under static conditions, the caveolin-1 distribution was much higher near the cell boundary than in the interior. However, the distribution changed dramatically at 24 h with most caveolin-1 distributed toward the middle of the cells (Fig. 6A and D).

Caveolin-1 is presented not only on the cytosolic face of the plasma membrane, but also in the cytoplasm and nucleus. To examine the detailed change in spatial distribution of caveolin-1 under shear stress, the original Z stack was split into apical and basal stacks (Fig. 7). In the basal stack, the local concentration of caveolin-1in the cell boundary was gradually disrupted with shear stress exposure time (Fig. 7A and 7E). Caveolin-1 presented a diffuse pattern, mostly localized in the interior of cells after 24 h of shear stress exposure (Fig. 7A and 7D), although the MFI and coverage of the basal stack were maintained at the static level at both 30 min and 24 h (Fig. 7B and C). In contrast, the MFI of caveolin-1 in the apical stack progressively increased with shear exposure time reaching significance relative to the static control and the 30 min time point after 24 h (Fig. 7B). The coverage of caveolin-1 in the apical stack was raised slightly, but not significantly relative to the static control after 24 h of shear stress exposure (Fig. 7C). Caveolin-1 did display a striking contrast in the MFI and coverage between the apical stack and basal stack at 24 h (151% vs. 91% in MFI, 87.1±6.1% vs. 68.0±12.8% in coverage, apical stack vs. basal stack, *P*<0.05). From this, we conclude that caveolin-1 was concentrated in the apical stack due to new synthesis and movement from the basal stack to the apical stack. Both in the apical and the basal stacks, the distribution of caveolin-1 after 30 min of shear stress exposure changed only slightly relative to the static control (Fig. 7D and E). After 24 h of shear stress exposure, the caveolin-1 distributed more in the cell interior and less near the boundary in the apical stack (Fig. 7D) and much less near the cell boundary in the basal stack (Fig. 7E).

### Redistribution of GM_1_ under Shear Stress

**Figure 8:**
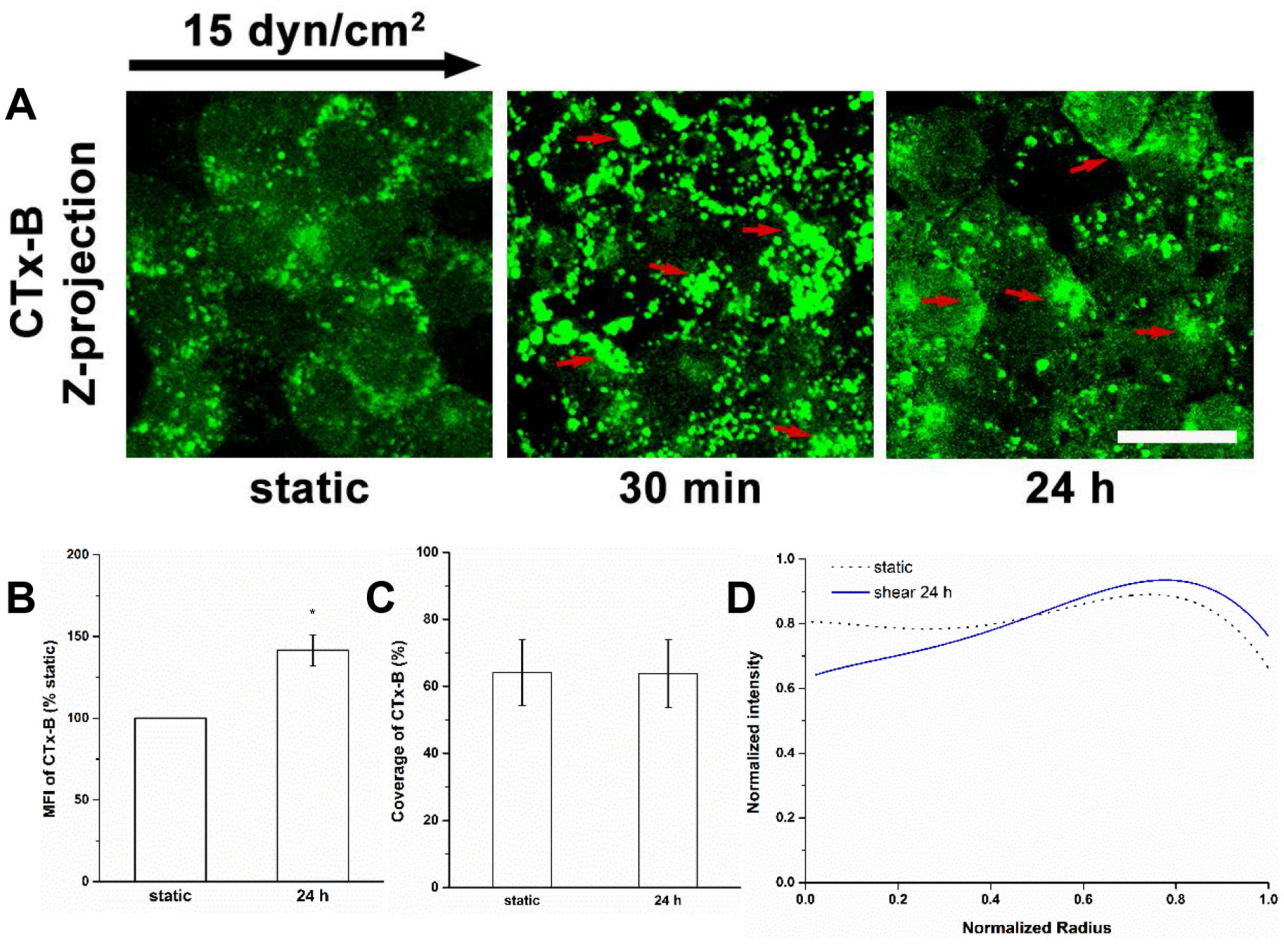
Redistribution of CTx-B labeled GM_1_ under shear stress. (A) The ganglioside GM_1_ was labeled with fluorescent CTx-B after shear stress exposure. The arrows indicate the clustering of GM_1_. (B) MFI, (C) Coverage, and (D) Radial distribution of CTx-B. The zero-radius represents the center of cell. GM_1_ was found to be clustered and recruited after 30 min of shear exposure [7]. The distribution of GM_1_ recovered close to the static level after 24 h, although much of GM_1_ was still clustered. Scale bar: 20 µm. *P<0.05.

We investigated the distribution of MRs by labeling the ganglioside GM_1_ with fluorescent CTx-B (Fig. 8). During shear application, GM_1_ clustered at the cell boundary (Fig. 8A and D). The MFI of CTx-B was significantly enhanced at 30 min (by about 31%), but there was no recruitment of caveolin-1 or glypican-1 to MR as shown by Western-blot assay in our previous study [7], suggesting the GM_1_ is recruited to the lipid raft fraction of MR. Only a small further increase in MFI at 24 h was observed (131% of static at 30 min vs. 142% of static at 24 h), indicating that the recruitment of GM_1_ was rapidly activated and then maintained at a nearly stable level.

The coverage of CTx-B labeled GM_1_ did not change with shear durations (Fig. 8C). Fig. 8D illustrates that the distribution of GM_1_ during shear exposure was time-dependent. The difference in distribution between the static condition and 30 min of shear exposure indicates that GM_1_ rapidly moved to the cell boundary under shear stress, similar to the movement of HS and glypican-1, as previously described [7]. The distribution of GM_1_ tended to return to the baseline distribution at 24 h. This may be associated with part of the newly synthesized glypican-1 (Fig. 4), and the extra labeling of GM_1_ in the immobile caveolae that accumulate near the center of the cell at 24 h (Fig. 7).

### The Spatial Distribution of Actin Cytoskeleton

**Figure 9:**
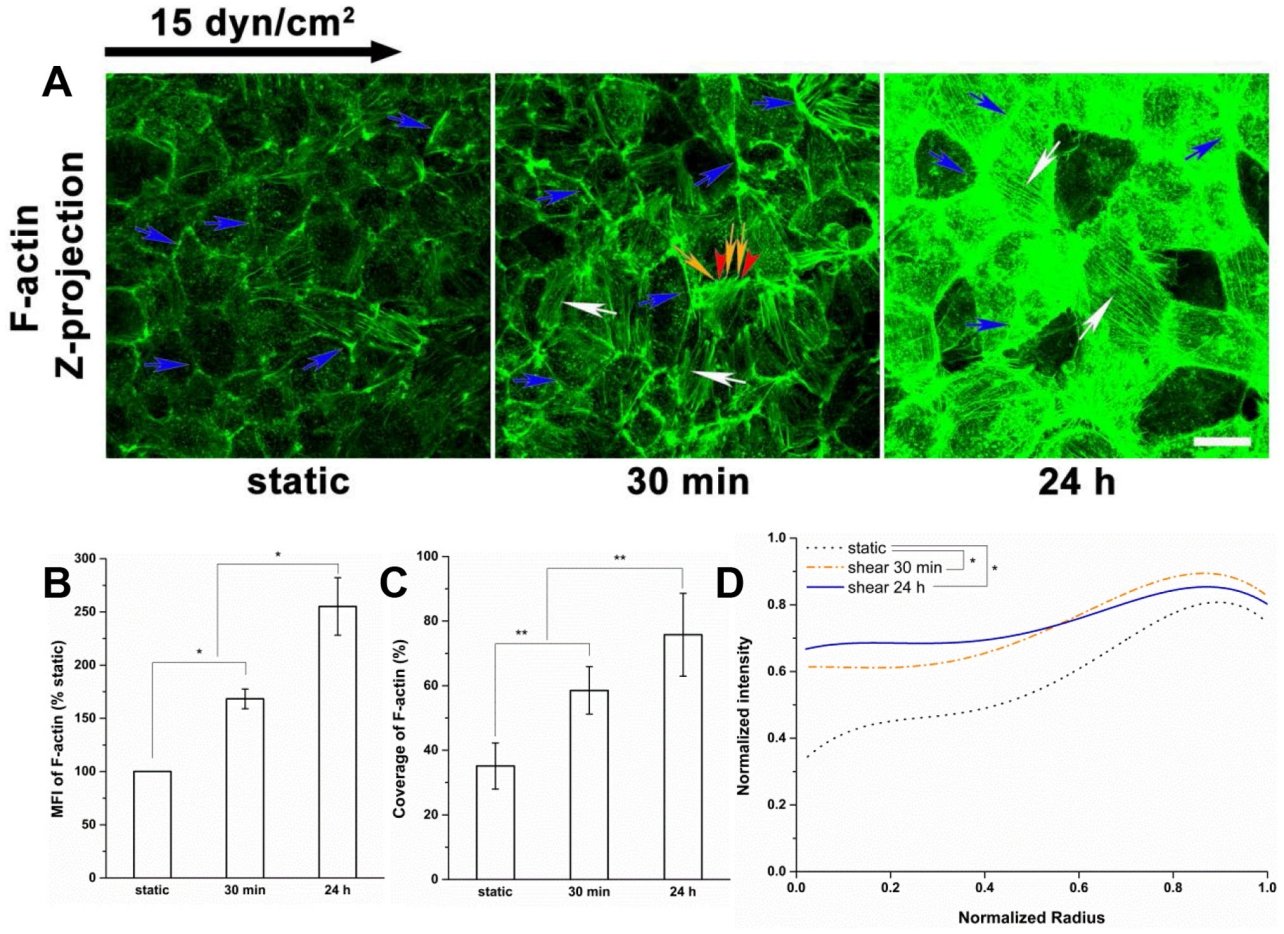
Redistribution of actin cytoskeleton under shear stress. (A) After flow application, the actin cytoskeleton was visualized with fluorescent phallotoxin. Blue arrows indicate the dense peripheral actin bands; white arrows indicate the stress fibers; and yellow arrows and red arrowheads denote the filopodia and lamellipodia, respectively. (B) MFI, (C) Coverage, and (D) Radial distribution of F-actin. F-actin was found most densely distributed along the edges of EC under static conditions, while fluid shear stress induced the polymerization of actin, the polarization of actin filaments (30 min), and the formation of stress fibers (24 h). Scale bar: 20 µm. *P<0.05; **P<0.01.

**Figure 10:**
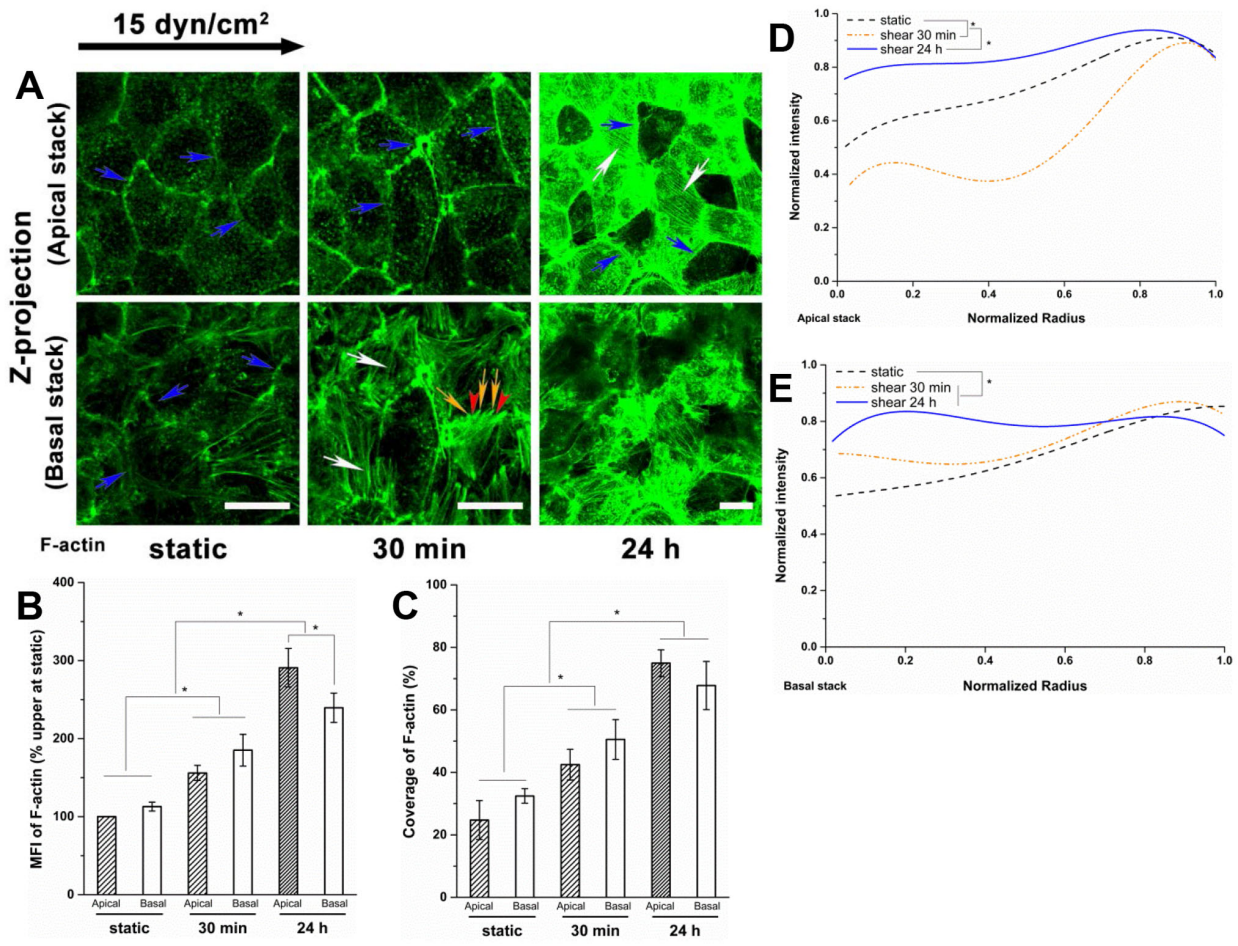
The vertical spatial distribution of actin cytoskeleton under shear stress. (A) F-actin in the apical and basal stack. The interface between two substacks is the plane crossing the center of the cell edges. Blue arrows indicate the dense peripheral actin bands; white arrows indicate the stress fibers; and yellow arrows and red arrowheads denote the filopodia and lamellipodia, respectively. (B) MFI, (C) Coverage, (D) radial distributions in the apical stack and (E) the basal stack. The polarized actin filaments were distributed slightly more in the basal stack than in the apical stack initially and after shear for 30 min. After exposure to shear stress for 24 h, the stress fibers were well assembled in the apical stack, but not the basal stack. In the apical stack, the actin distribution was still concentrated near the cell boundary after 30 min of shear exposure, and became more uniform after 24 h, compared to the static conditions; in the basal stack, the distribution was more uniform at all times. Scale bar: 20 µm. *P<0.05.

The dynamics of the spatial redistribution of actin in response to shear stress was studied (Figs. 9 and 10). Consistent with other EC types, such as BAECs [22], [24], [26], the dense peripheral actin bands were present under static conditions at the cell periphery of RFPECs (Fig. 9A). In response to shear stress for 30 min, the polymerization and polarization of actin filaments were obvious; stress fibers were oriented preferentially parallel to the nearest edge; and lamellipodia and filopodia emerged. The polymerization and polarization of actin filaments on cells exposed to 24 h of shear stress were further strengthened as prominent stress fibers were observed (Fig. 9A). By quantitatively analyzing the changes of the actin cytoskeleton, we found that the MFI and coverage of F-actin increased dramatically with shear duration (Fig. 9B and C), and the distributions of F-actin after shear application became much more uniform compared to the static controls, indicating that the increased stress fibers were spread across the cell and not concentrated at the cell boundary (Fig. 9D).

The apical and basal stacks were used to further examine the spatial characteristics of the actin cytoskeleton distribution (Fig. 10). In static cells, we found that dense peripheral bands were visible on both the apical and basal stacks (Fig. 10A). In the apical stack, a stronger intensity of dense peripheral bands was present at 30 min and 24 h compared to the static controls, but numerous long stress fibers became evident only at 24 h. In contrast, the dense peripheral bands in the basal stack were dispersed and gradually not distinguishable with increasing shear durations. Nevertheless, stress fibers, lamellipodia and filopodia protrusions emerged at 30 min, but by 24 h F-actin was scattered and arranged in a disorderly and irregular fashion in the basal stack (Fig. 10A). The actin in the apical stack was concentrated near the cell boundary under static conditions, further enhanced in the cell boundary after shear stress exposure for 30 min, and significantly increased in the cell interior resulting a nearly uniform distribution after 24 h (Fig. 10D). The actin in the basal stack progressively increased in the cell interior with shear stress duration rapidly reaching significance relative to the static control after 30 min (Fig. 10E). Correspondingly, the MFI and coverage of F-actin increased significantly with shear duration in both apical and basal stacks (Fig. 10B and C), indicating strong biological activity (synthesis) in both the apical and basal aspects of the cell. Notably, the MFI of F-actin at 24 h in apical stacks was greater than that in the basal stacks (about 291% vs. 240% of static, in the apical vs. basal stacks, *P*<0.05).

### Role of the Actin Cytoskeleton in Reorganization of the Glycocalyx

**Figure 11:**
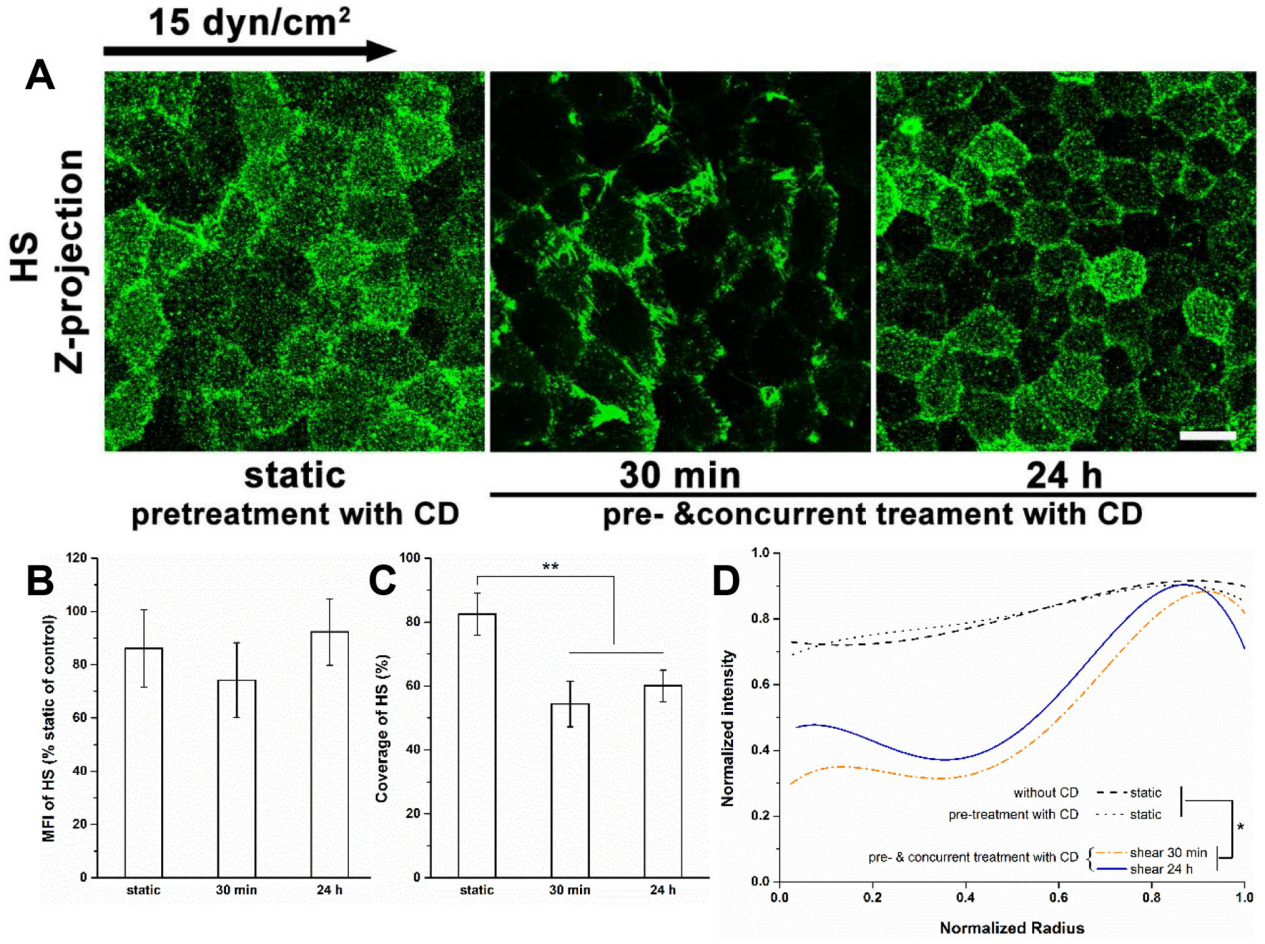
Redistribution of HS in the presence of cytochalasin D (CD). Cells were treated with CD, an inhibitor of actin polymerization, 1(A) Confocal images, (B) MFI, (C) Coverage, and (D) Radial profile of HS in the presence of CD. Disruption of the actin cytoskeleton by CD did not influence HS under static conditions and after shear exposure for 30 min, but attenuated the shear stress-induced recovery of HS at 24 h. The distribution of HS at 24 h in the presence of CD was close to the level at 30 min. Scale bar: 20 µm. **P<0.01.

To further validate the importance of actin remodeling in the reorganization of the glycocalyx, we measured the dynamics of HS under shear stress with cells treated with cytochalasin D to disrupt actin reorganization (Fig. 11, compare to Fig. 1 without cytochalasin D). It is clear that the re-coverage of HS at 24 h is blocked (panels A, C and D) as well as the new synthesis of HS (panel B).

## Discussion

Our laboratory previously investigated the role of the glycocalyx in mechanotransduction at short times [8] and the early response of the glycocalyx to fluid shear stress [7]. Those studies corresponded to the time frame of many in vitro investigations of EC mechanotransduction [35], [36] and endothelial permeability [37], [38]. It was demonstrated that the selective enzymatic removal of GAG components, such as HS, results in the complete inhibition of shear stress-induced nitric oxide (NO) production [39], [40], and that the glycocalyx is shear sensitive and closely linked to membrane rafts and transmembrane structures. During the initial 30 min of shear exposure, the movement of HS is predominantly associated with glypican-1 that is anchored to mobile lipid rafts, whereas the glypican fraction linked to caveolae is not mobile; the immobility of CS and the remainder of HS is associated with the transmembrane protein syndecan-1 [7]. The present study revealed the adaptation of the glycocalyx to shear stress during 24 h of shear exposure that should more faithfully represent the condition of fully adapted endothelial cells in vivo.

Several studies have detected a substantial glycocalyx on cultured RFPECs, the main cell type employed in the present study [28], [31], [41]. We used RFPECs as a model cell for visualizing the response of the glycocalyx to shear stress since these cells display several characteristic endothelial mechanoresponses including intercellular and cytoskeleton junction remodeling [28] and shear-induced NO production, and are immuno-reactive to a wide range of glycocalyx component antibodies [7].We used the often studied BAECs to confirm the dramatic change in HS distribution. Similar phenomena occurred on RFPECs and BAECs showing consistent changes in the synthesis and reorganization of HS (Figs. 1 and 2).

We also developed a new scattering distribution analysis method for BAECs after 24 h of exposure because they remodeled into an elongated (fusiform) shape (Fig. 2) whereas RFPECs retained their cobblestone morphology at 24 h (Fig. 1).Other EC types as well do not elongate in response to sustained shear stress. For example, during exposure to 40 dyn/cm^2^ for 24 h, pig aortic ECs did not align along flow direction [42]. The BAECs did maintain cobblestone morphology after 30 min of shear exposure. To compare the radial distribution and scattering distribution methods on cobblestone cells, we measured the HS distribution on BAECs under both static and shear (30 min) conditions, and observed that both methods gave nearly the same distributions for cobblestone ECs (Fig. 2D). Therefore, we used radial distribution analyses for RFPECs at all time points since they retained a cobblestone morphology.

The distributions of HS and glypican-1 became nonuniform after 30 min of shear exposure (clustering at the cell boundary), and then returned to a nearly uniform distribution between 30 min and 24 h (Figs. 1, 2 and 4). The distributions of CS and syndecan-1 were not altered throughout the duration of shear exposure (Figs. 3 and 5). The in vivo state was examined in [43] where it was shown that the fully adapted state in the aorta of rats and mice shows a highly uniform coverage of HS that is similar to our 24 h state. Other gylcocalyx components have not yet been examined in vivo.

At 30 min we further observed reductions in HS and glypican-1 coverages without MFI changes, and no alterations in coverage and MFI of syndecan-1 with bound CS. The decreases in the coverages and altered distributions of HS and glypican-1 are clearly due to the movement of glypican with anchored HS toward the cell’s downstream edge. At 24 h, the coverages of HS and glypican-1 were restored to their static levels (Figs.1, 2 and 4), that of CS remained at its static level (Fig. 3) and that of syndecan-1 increased significantly (Fig. 5); the MFIs of all glycocalyx components were increased above their static levels. However, the boundary clustering of HS and glypican-1 were still clearly visible. We conclude from the increased MFIs that the glycocalyx components were synthesized during shear exposure for 24 hours. This is consistent with other studies in pig aortic EC [42] and human EC-RF24 cells [44] showing that shear stress induces new synthesis of HS and CS. Recently, Koo et al [45] examined the effect of pulsatile flow on glycocalyx formation in cultured human umbilical vein ECs (HUVECs). They reported that their atheroprotective waveform (high mean shear, no reversal) induced increases in HS and syndecan-1, a decrease in glypican-1, and no alteration of CS after 7 days of exposure. Another study showed that glypican-1 did not change on HUVECs exposed to the atheroprotective waveform for 3 days [46].

The altered distributions of glycocalyx components indicate reorganization of membrane microdomains. The reduction of caveolin-1 in the cell boundary in the basal stack and the increase in the cell interior in the apical stack at 24 h are prominent characteristics of the reorganization of caveloin-1 over time (Figs. 6 ad 7). The increased MFI and coverage in the apical stack compared to the basal stack at 24 h indicates increased caveolae in the central region of the apical membrane (Fig. 7). This is consistent with previous work showing the assembly of caveolae on the apical membrane in ECs exposed to shear flow for 6 h [47].

The shear stress-induced increase in caveolae in the apical membrane after 24 h of shear exposure (Fig. 7) appears to be associated with the newly synthesized HS and glypican-1 and their distributions (Figs. 1, 2 and 4). The distribution of GM_1_ is still skewed toward the boundary (Fig. 8) indicating that the clustering of mobile lipid rafts with anchored glypican-1 and HS is still present after 24 h.

Because the actin cytoskeleton interacts with the transmembrane core protein syndecan-1 and the caveolar structural protein caveolin-1 for stabilization, it was visualized in the present study (Figs. 9 and 10). Although the RFPEC did not elongate significantly after 24 h of shear stress exposure (Fig. 1A), the formation of stress fibers running along the cellular axis was apparent (Fig. 9A). Similar results have been found on other EC types including BAEC [22], [24], [26]. In RFPECs, dense peripheral bands were present in static cells; lamellipodia and filopodia protrusions and stress fibers emerged after shear exposure of 30 min and prominent stress fibers were observed at 24 h (Fig. 10A).

We also present findings that have not been observed previously - notably the shear stress-induced time-dependent spatial distribution of the actin cytoskeleton. Fig. 10A displays the detailed changes in spatial distribution, which present the z-projection images of substacks (z-projection of original stack was shown in Fig. 9A). Shear stress induced F-actin was distributed in both the apical and basal stacks (Fig. 9B–C and 10B–C). Long stress fibers were formed mainly in the apical stack after 24 h of shear exposure (Fig. 10A). The dense peripheral bands were dispersed at 30 min in the basal stack, but were still prominent at 30 min and detectable at 24 h in the apical stack. The F-actin appeared disorderly and irregular without clear lamellipodia and filopodia in the basal stack at 24 h (Fig. 10A). The shear-induced stress fibers were distributed more prominently in the central region of the cell relative to the cell boundary at 24 h compared to static or 30 min (Fig. 9D, 10D and E). The actin in the central region of the cell progressively increased in the basal stack with shear duration, and dramatically increased in the apical stack after 30 min, suggesting that actin-mediated mechanotransduction of shear stress is time-and space- dependent. Interestingly, the administration of CD (40 nM) did not prevent the clustering of HS in response to shear stress at 30 min, but abolished the re-coverage of HS at 24 h (Fig. 11), suggesting that the shear stress-induced clustering of HS at 30 min is actin cytoskeleton-independent, and that the actin cytoskeleton plays an important role in the re-organization of the glycocalyx at 24 h.

The associations of syndecan-1 with stress fiber and lamellipodia protrusion have been indicated in several studies [10], [48], [49]. Stress fibers are believed to support caveolae in the apical membrane by their association with caveolin-1 [17], [19]. Thus it appears that the distribution of F-actin over the cell surface, including that which has been newly synthesized, provides a supporting scaffold for new caveolae and their associated glypican/HS. It has been demonstrated that caveolae and caveolin-1 are crucial for both short- and long-term mechanotransduction in blood vessels of mice [50]. Newly synthesized syndecan supported by actin provides a platform for additional HS and CS that have been synthesized as well.

**Figure 12:**
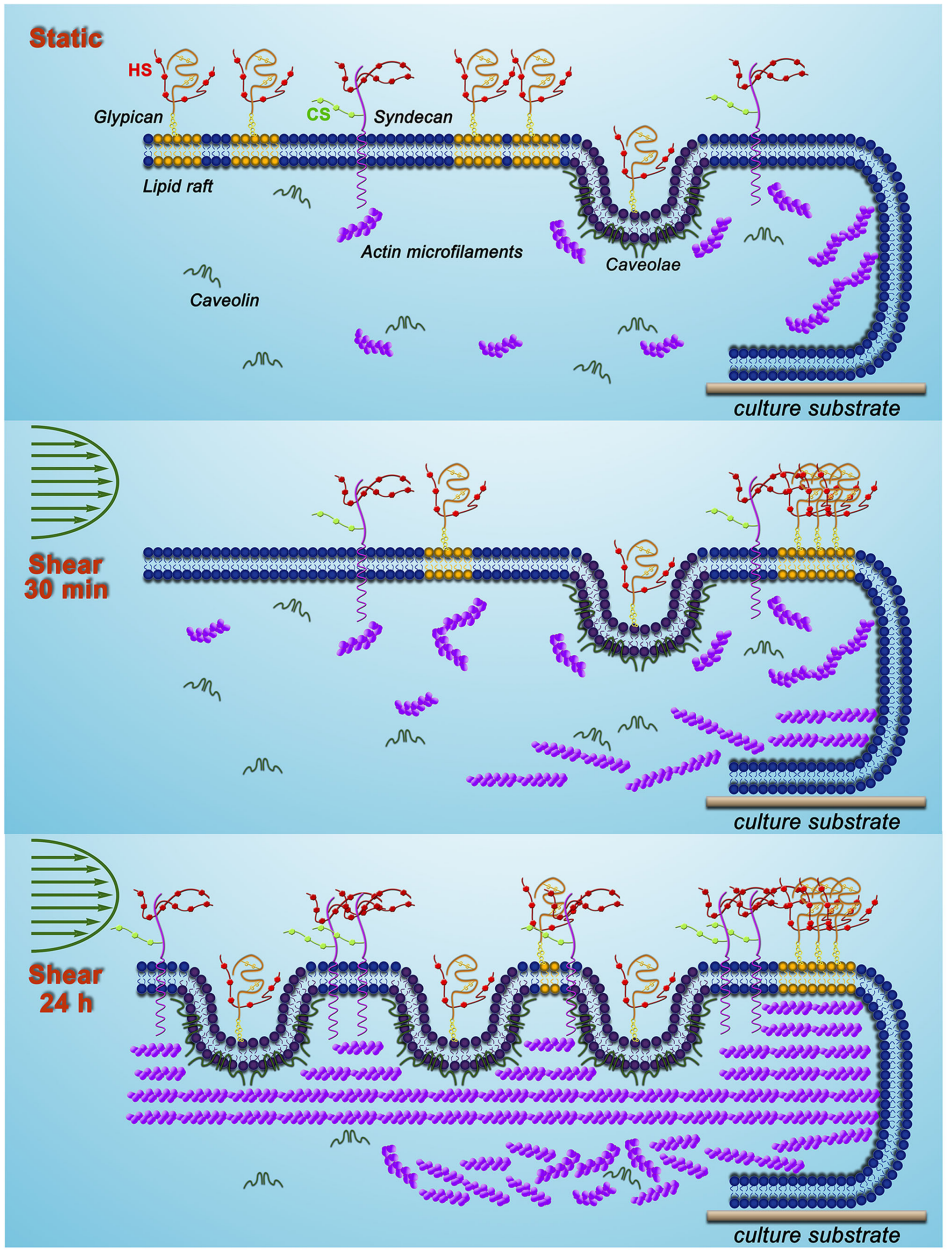
Adaptive remodeling of glycocalyx with membrane rafts and actin cytoskeleton. **Under static conditions,** glypican-1carrying only HS is localized on the dispersed lipid rafts and caveolae on the membrane. The actin cytoskeleton interacts with the transmembrane protein syndecan-1 and the caveolar structural protein caveolin-1 for stabilization. **After 30 min of shear exposure**, lipid rafts have carried glypican-1 with anchored HS to the cell boundary (clustering), while syndecan-1 carrying HS and CS, and caveolae with localized glypican-1 and anchored HS, do not move. Actin microfilaments increase in both apical and basal aspects of the cell. **After 24 h of exposure**, new caveolae are assembled on the apical surface, which may associate with newly synthesized glypican-1. Syndecan-1 (HS/CS), and glypican-1(HS) that is bound to anchored caveolae, and mobile lipid rafts are synthesized and result in nearly uniform distributions of HS and CS. Numerous long stress fibers form and most distribute in the apical part of the cell, where they stabilize new caveolae and syndecan-1. In the basal part of the cell, actin microfilaments increase, scatter and arrange in a disorderly fashion. Our findings portray a dynamic reorganization of the EC glycocalyx.

Figure 12 illustrates the adaptive remodeling of the glycocalyx, the associated membrane rafts and the actin cytoskeleton as described in detail in the figure caption. The adaptation of the glycocalyx to fluid shear stress involves a balance between the synthesis of glycocalyx components including both GAGs and core proteins, and their degradation that is modulated by enzymes such as heparinase and metalloproteases [4], [6]. Increases in sulfated GAGs in the circulation media were detected after 24 h of shear exposure in an earlier study [42]. Although we did not observe enhanced sulfated GAGs in the circulation media after 30 min of shear exposure in a previous study [7], we have not examined this issue at 24 h due to loss of cells from the edges of the cover slide artificially elevating the media concentration of sulfated GAG.

Nonetheless, there was a clear enhancement of all GAGs and core proteins after 24 h of shear as indicated by significant increases in MFI. Notably, the mechanisms underlying the shear stress-induced increase in GAG synthesis are still not known. GAG synthesis induced by shear stress was concomitant with a decrease in DNA synthesis and an increase in protein synthesis [42]. The mRNA expressions of exostosin glycosyltransferase-1 and -2 (EXT1 and EXT2), two genes encoding glycosyltransferases involved in the chain elongation step of HS biosynthesis, did not change under shear stress [45]. The disruption of actin cytoskeleton by CD abolished the additional synthesis of HS on ECs exposed to shear stress for 24 h (Fig. 11), indicating that the actin cytoskeleton plays a role in shear-induced HS biosynthesis.

The new synthesis of HS and CS in EC exposed to shear stress has been observed in several studies. Arisaka et al. [42] detected the synthesis of sulphated GAGs in pig aortic EC exposed to shear stresses of 15 or 40 dyn/cm^2^ for more than 24 h. Gouverneur et al. [44] demonstrated the enhancement of HA and sulfated GAGs on human EC-RF24 cells exposed to 9.7 dyn/cm^2^ of shear stress for 24 h. Our findings focused on the sulfated GAGs and revealed that both HS and CS were synthesized on RFPECs exposed to 15 dyn/cm^2^ of shear stress for 24 h (Figs. 1–3), but not for 30 min [7]. In addition, the major carriers of GAGs, syndecan-1 and glypican-1 increased at 24 h (Figs. 4 and 5) but not 30 min [7]. The MFIs of HS, CS, syndecan-1 and glypican-1were raised by 43%, 20%, 63% and 50%, respectively, compared to static conditions (Figs. 1–4). The large increases in MFI and coverage of both syndecan-1 and glypican-1 suggest that more HS than CS is synthesized because syndecan-1 binds both HS and CS chains while glypican-1 is exclusively linked to HS. Our findings portray a dynamic reorganization of the glycocalyx, associated membrane rafts and actin cytoskeleton that may underlie alterations in endothelial mechanotransduction mechanisms over the time course of shear exposure.

### Pathophysiological Implications of the Adaptation of the Glycocalyx to Shear Stress

EC in large arteries are responsive to their fluid shear stress environment, taking on an elongated, shear-aligned morphology in atheroprotected regions and a cobblestone, non-aligned morphology in atheroprone regions [51]. Cultured EC display the atheroprone phenotype under static conditions and short exposures to shear stress whereas they display the atheroprotected phenotype after 24 h of exposure to moderate shear stress levels. The present study (24 h shear exposure) and our recent study (30 min shear exposure) [7] reveal dramatic differences in glycocalyx organization that may underlie differences in mechanotransduction mechanisms as well as the selective permeability and leuckocyte adhesion barrier properties in atheroprone and atheroprotected regions of the circulation. These differences in glycocalyx structure may be relevant to the underlying causes of many pathologies including stroke, hypertension and diabetes [51]–[53].
